# Mitotic catastrophe is a putative mechanism underlying the weak correlation between sensitivity to carbon ions and cisplatin

**DOI:** 10.1038/srep40588

**Published:** 2017-01-16

**Authors:** Daijiro Kobayashi, Takahiro Oike, Atsushi Shibata, Atsuko Niimi, Yoshiki Kubota, Makoto Sakai, Napapat Amornwhichet, Yuya Yoshimoto, Yoshihiko Hagiwara, Yuka Kimura, Yuka Hirota, Hiro Sato, Mayu Isono, Yukari Yoshida, Takashi Kohno, Tatsuya Ohno, Takashi Nakano

**Affiliations:** 1 Department of Radiation Oncology, Gunma University Graduate School of Medicine, Maebashi, Gunma, Japan; 2 Division of Genome Biology, National Cancer Center Research Institute, Chuo-ku, Tokyo, Japan; 3 Advanced Scientific Research Leaders Development Unit, Gunma University, Maebashi, Gunma, Japan; 4 Research Program for Heavy Ion Therapy, Division of Integrated Oncology Research, Gunma University Initiative for Advanced Research, Maebashi, Gunma, Japan; 5 Gunma University Heavy Ion Medical Center, Maebashi, Gunma, Japan; 6 Department of Radiology, Chulalongkorn University, Pathumwan, Bangkok, Thailand

## Abstract

In cancer therapy today, carbon ion radiotherapy is used mainly as monotherapy, whereas cisplatin is used concomitantly with X-ray radiotherapy. The effectiveness of concomitant carbon ions and cisplatin is unclear. To obtain the information on the mechanisms potentially shared between carbon ions or X-rays and cisplatin, we assessed the correlation of sensitivity to the single treatments. In 20 human cancer cell lines, sensitivity to X-rays strongly correlated with sensitivity to cisplatin, indicating the presence of potentially shared target mechanisms. Interestingly, the correlation of sensitivity to carbon ions and cisplatin was much weaker than that of sensitivity to X-rays and cisplatin, indicating the presence of potentially different target mechanisms between carbon ions and cisplatin. Assessment of clonogenic cell death by 4′,6-diamidino-2-phenylindole dihydrochloride staining showed that mitotic catastrophe was more efficiently induced by carbon ions than by the same physical dose of X-rays, while apoptosis and senescence were not. These data indicate that the correlation of sensitivity to carbon ions and cisplatin is weaker than that of sensitivity to X-rays and cisplatin, which are helpful as biological basis to understand the potentially shared mechanism among these treatments. Further investigation is mandatory to elucidate the clinical efficacy of carbon ions and cisplatin combination.

Carbon ion radiotherapy is an emerging cancer therapy that has two advantages over X-rays: a sharp dose distribution and a strong cell killing effect^1^. Clinical experience shows that carbon ion radiotherapy has a promising anti-tumor effect^2^,^3^. Carbon ion radiotherapy is currently at the developmental stage, and the maximum tolerable doses are being examined in clinical dose-escalation trials^2^,^3^. For this reason, carbon ion radiotherapy is mainly utilized as a monotherapy. By contrast, co-treatment with conventional X-ray radiotherapy and chemotherapy is common^4^. Cisplatin is one of the chemotherapeutic drugs most commonly used in combination with X-rays. Clinical evidence shows that addition of cisplatin increases the efficacy of X-ray radiotherapy^5^,^6^. Thus, it is possible that concomitant use of cisplatin will also increase the efficacy of carbon ion radiotherapy. However, preclinical data regarding the combined effect of carbon ions and cisplatin are limited; only a few small-scale studies have been conducted (Supplementary Table 1)^7^,^8^,^9^,^10^. Data from these studies are unclear as to whether the cell killing effect of carbon ions plus cisplatin is synergistic.

The combined effect of X-rays and cisplatin has been extensively studied in the preclinical setting; however, there are no firm conclusions as to whether the effects are synergistic or purely additive because results vary widely from marked synergism to none at all (Supplementary Table 1)^8^,^9^,^10^,^11^,^12^,^13^,^14^,^15^,^16^,^17^,^18^,^19^,^20^,^21^. Furthermore, the presence/absence of synergism is inconsistent even between studies using the same cell lines (i.e., Agoni *et al. vs.* Flentje *et al*. for HeLa cells; Rava-Frank *et al. vs.* Liu *et al*. for A549 cells; and Rava-Frank *et al. vs.* Flentje *et al*. for CaSki cells)^12^,^13^,^18^,^19^. These data suggest methodological limitations of these studies with respect to the assessment of cell killing: synergism was assessed by comparing linear-quadratic dose-response curves after treatment with mono- and combined modalities. In fact, this limitation has been pointed out by multiple groups over decades^22^,^23^,^24^. In addition, the small number of cell lines tested in most studies (11/14 studies used <3 cell lines) makes it difficult to draw firm conclusions on this issue (Supplementary Table 1). These data strongly suggest the difficulty of examining the efficiency of the combination of carbon ions and cisplatin in terms of cell killing by using the same methods.

With taking these issues into account, here we adopted a different approach. In order to obtain the information on the mechanisms potentially shared between carbon ions or X-rays and cisplatin, we assessed the correlation of sensitivity to the single treatments in 20 human cancer cell lines. Although experiments utilizing combined treatment are mandatory to elucidate the clinical efficacy of combination treatment, the correlation analysis for sensitivity to single treatments will be a biological basis to understand the potentially shared mechanism among these treatments.

## Results and Discussion

**Figure 1:**
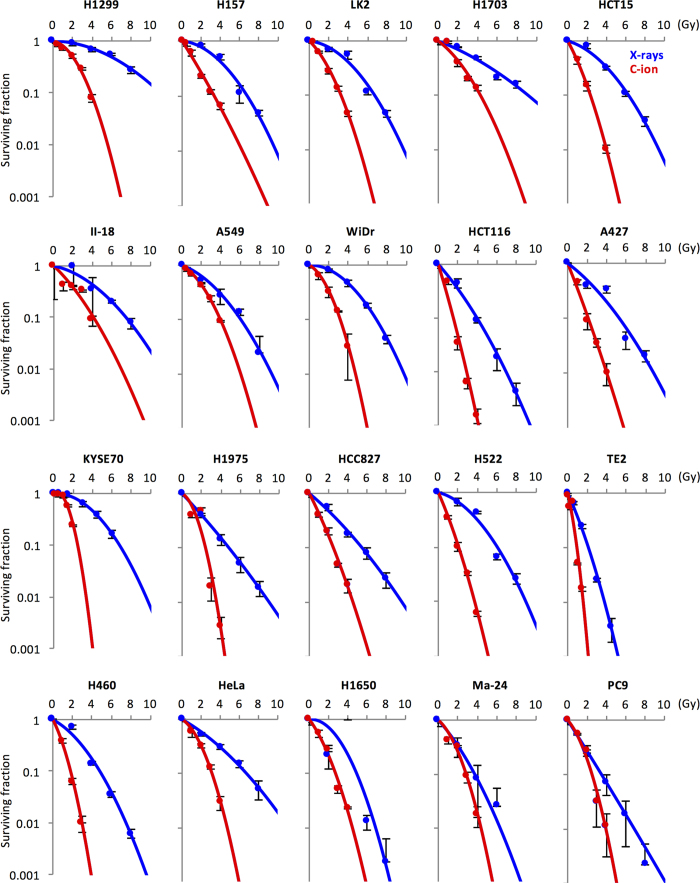
Sensitivity of cancer cells to X-rays or carbon ions. Cells were irradiated with X-rays or carbon ions and subjected to a clonogenic survival assay. Experiments were performed at least in triplicate. Data are presented as the mean ± standard deviation. The survival curves were fitted to the linear-quadratic model. C-ion, carbon ion.

**Figure 2:**
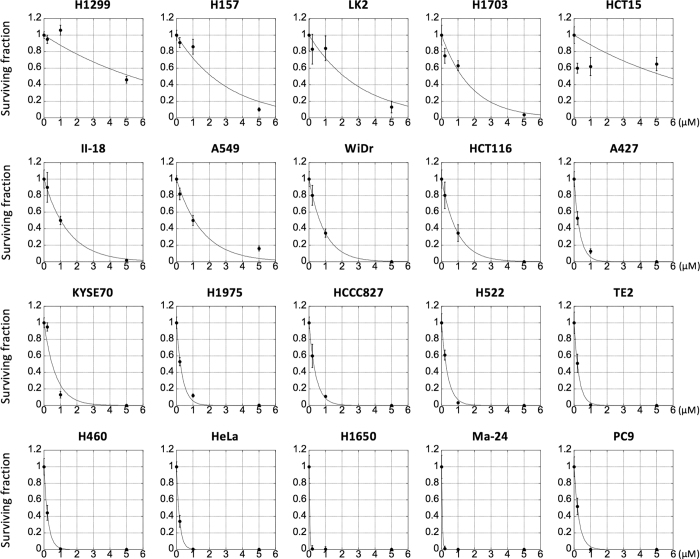
Sensitivity of cancer cells to cisplatin. Cells were exposed to cisplatin for 24 h and subjected to a clonogenic survival assay. Experiments were performed at least in triplicate. Data are presented as the mean ± standard deviation. The survival data were fitted using an exponential function.

We examined the sensitivity of 20 human cancer cell lines to X-rays, carbon ions, or cisplatin in a clonogenic survival assay (Figs 1,2). Details of the parameters used to assess radiation sensitivity are summarized in Supplementary Table 2. We then examined the correlation of sensitivity to X-rays and cisplatin as well as that of sensitivity to carbon ions and cisplatin using Spearman’s Rank Order test. Previous studies show that it is difficult to assess the efficiency of the combined effects of X-rays and cisplatin by comparing only a few dose points^22^,^23^,^24^. Therefore, we performed a comprehensive correlation analysis using the following indices of treatment sensitivity and sought to obtain an overall trend: (i) the surviving fraction at all examined dose points, except 5 μM cisplatin (i.e., 2, 4, 6, and 8 Gy for X-rays, 1, 2, 3, and 4 Gy for carbon ions, and 0.2 and 1 μM for cisplatin). The surviving fraction at 5 μM cisplatin was not used because it was <0.01 in 70% of the cell lines examined, and was thus considered inappropriate for correlation analysis; (ii) the D_10_ value for X-rays and carbon ions; and (iii) the IC_50_ value for cisplatin. The D_10_ value is a standard index of radiation sensitivity that represents the dose yielding a surviving fraction of 10%, whereas the IC_50_ value is a standard index for drug sensitivity that represents a dose yielding a surviving fraction of 50%^25^,^26^.

**Figure 3:**
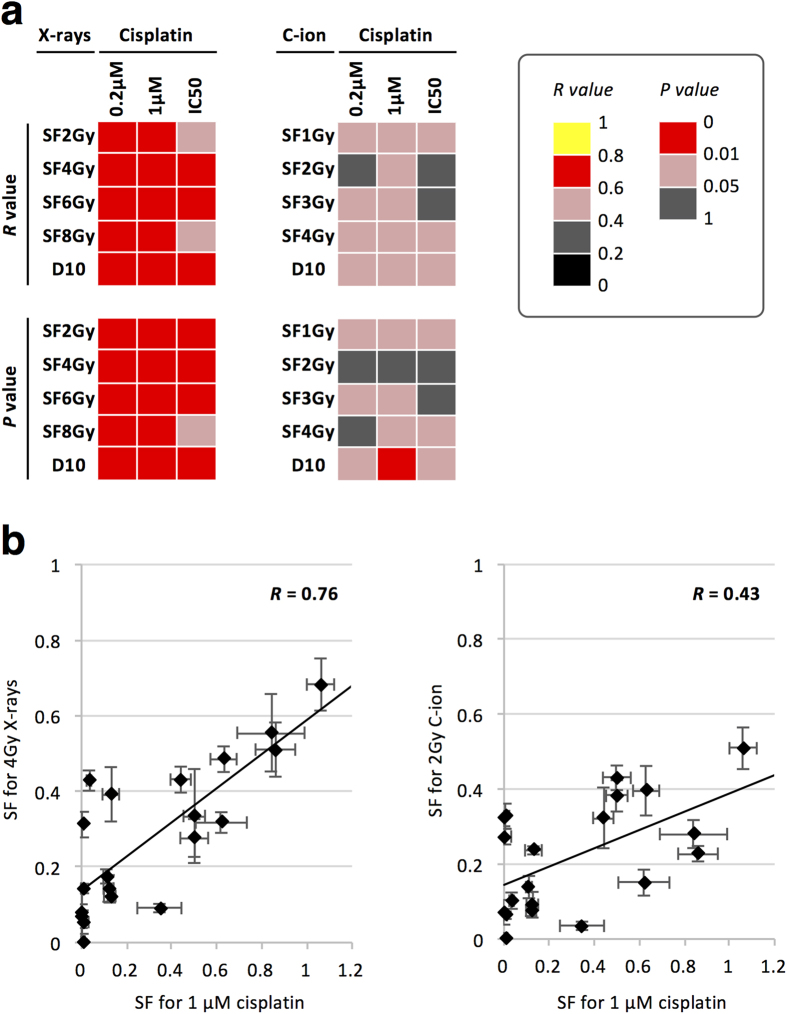
Correlation of the sensitivity of cancer cells to X-rays or carbon ions with that to cisplatin. **(a)** Strength (*R* value) and statistical significance (*P* value) of the correlation between X-ray and cisplatin sensitivities (left panel) and that of the correlation between carbon ion and cisplatin sensitivities (right panel) as assessed by Spearman’s Rank Order test. **(b)** Typical example for X-rays and cisplatin (left panel) and between carbon ions and cisplatin (right panel). Data are expressed as the mean ± standard deviation. C-ion, carbon ion; SF, surviving fraction. SFXGy indicates the SF at X Gy.

The correlation of sensitivity to X-rays and cisplatin was statistically significant at all 15 endpoints tested (Fig. 3, Supplementary Table 3). The correlation was strong (i.e., *R *> 0.6) at 87% (13/15) of the endpoints. These data indicate that X-rays and cisplatin have potentially shared target mechanisms with respect to cell killing. Interestingly, carbon ion and cisplatin sensitivities showed a statistically less significant correlation than X-ray and cisplatin sensitivities, i.e., *P *< 0.05 at 67% (10/15) and 100% (15/15) of the endpoints, respectively, and *P *< 0.01 at 6.7% (1/15) and 93% (14/15) of the endpoints, respectively (Fig. 3, Supplementary Tables 3,4). Accordingly, carbon ion and cisplatin sensitivities were more weakly correlated than X-ray and cisplatin sensitivities, i.e., *R *> 0.6 in 0% (0/15) and 87% (13/15) of the endpoints, respectively. These data indicate that carbon ions and cisplatin have potentially different target mechanisms with respect to cell killing.

**Figure 4:**
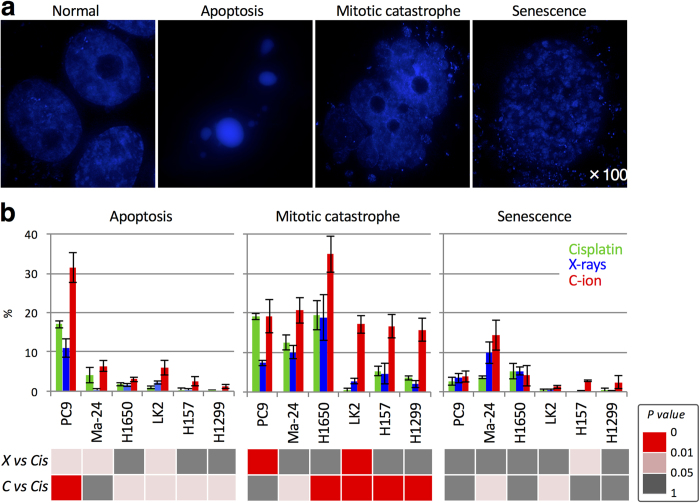
Clonogenic cell death induced by cisplatin, X-rays, or carbon ions. Cells were treated with X-rays (4 Gy), carbon ions (4 Gy), or cisplatin (1 μM for 24 h) and then stained with DAPI 72 h later. Apoptosis, mitotic catastrophe, and senescence were determined according to characteristic nuclear morphologies (see “Materials and Methods” for definitions). **(a)** Representative images showing the nuclear morphology of normal cells and cells undergoing apoptosis, mitotic catastrophe, and senescence. Images of untreated Ma-24 cells (normal) and cells treated with cisplatin (undergoing apoptosis, mitotic catastrophe, or senescence) were taken using a DeltaVision (GE Healthcare, Little Chalfont, UK) and deconvoluted and processed using SoftWoRx (GE Healthcare)^35^. **(b)** Mode of clonogenic cell death in cells treated with X-rays or carbon ions plus cisplatin. In the lower panels, the statistical significance of differences in the levels of apoptosis, mitotic catastrophe, or senescence induced by X-rays (X) or carbon ions (C) plus cisplatin (Cis) is indicated by different colors.

Next, we sought to investigate the cellular mechanisms that contribute to the weaker correlation of sensitivity to carbon ions and cisplatin than that of sensitivity to X-rays and cisplatin. We focused on clonogenic cell death (i.e., apoptosis, mitotic catastrophe, and senescence) because it is the final endpoint of a cellular response to such treatments. Apoptosis, mitotic catastrophe, and senescence were examined by morphologic observation of 4′,6-diamidino-2-phenylindole dihydrochloride (DAPI)-stained nuclei (Fig. 4a)^27^,^28^,^29^,^30^,^31^. PC9, Ma-24, and H1650 cells that are highly sensitive to cisplatin, and H1299, H157, and LK2 cells that are resistant to cisplatin, were used. In all six cell lines, the total amount of apoptosis, mitotic catastrophe, and senescence showed a strong correlation with the surviving fraction as measured by the clonogenic survival assay (Supplementary Fig. 1). These data indicate the validity of the DAPI staining assay for assessing clonogenic cell death. In addition, the fraction of X-ray- or carbon ion-irradiated PC9 cells undergoing apoptosis and mitotic catastrophe as assessed by DAPI staining showed fair agreement with the fractions of annexin-V (AV)-positive cells and AV-negative/propidium iodide (PI)-positive cells assessed by flow cytometry (Supplementary Fig. 2). AV is a marker of apoptosis (and subsequent necrosis), whereas PI is a marker of non-viable cells^32^,^33^. Taken together, these data support the robustness of the DAPI staining assay for distinguishing between apoptosis and mitotic catastrophe.

When comparing cells treated with X-rays (4 Gy) and cisplatin (1 μM), the differences in the levels of apoptosis, mitotic catastrophe, and senescence were modest and were not statistically significant in 50%, 67%, and 83% of the cell lines tested, respectively (Fig. 4b). When comparing cells treated with carbon ions (4 Gy) and cisplatin (1 μM), the difference in the level of apoptosis was statistically significant in 83% of the cell lines tested; however, the difference was modest (2.3%, 1.2%, 5.0%, 2.1%, and 1.1% in Ma-24, H1650, LK2, H157, and H1299 cells, respectively), with the exception of PC9 cells, which showed a difference of 14%. The difference in the level of senescence was also modest between cells treated with carbon ions and cisplatin; the difference was not statistically significant in 67% of the cell lines tested. Interestingly, when comparing cells treated with carbon ions and cisplatin, the difference in the level of mitotic catastrophe was more evident than the differences in the levels of apoptosis and senescence; the difference in the level of mitotic catastrophe was statistically significant in 83% of the cell lines tested (8.1%, 15%, 17%, 11%, and 12% in Ma-24, H1650, LK2, H157, and H1299 cells, respectively), which was greater than the difference between the level of apoptosis and that of senescence. Together, these data indicate that apoptosis and senescence may be shared mechanisms for cell killing among carbon ions, X-rays and cisplatin, whereas mitotic catastrophe may be a mechanism that is differentially triggered by carbon ions.

Previous studies suggest that the putative mechanism underlying the efficient induction of mitotic catastrophe by carbon ions is as follows. Cells exposed to ionizing radiation enter G2/M cell cycle arrest, which allows repair of DNA double-strand breaks (DSBs). However, cells are released from G2/M arrest within a few days after irradiation with carbon ions and X-rays^34^. This is due to the limitation of the G2/M checkpoint machinery, i.e., a cell is released from arrest when the number of DSBs becomes lower than ∼10–20^35^,^36^. Following G2/M checkpoint release, cells harboring 10–20 DSBs are able to enter mitosis^37^,^38^. Mitotic catastrophe occurs through aberrant mitosis due to unrepaired DSBs^39^. DSBs induced by carbon ions are repaired significantly less effectively than those generated by X-rays^34^. The low repair efficacy of carbon ion-induced DSBs can be attributed to their structural complexity^40^,^41^,^42^. It is likely that the difficulty in repairing complex DSBs induced by carbon ions contributes to the efficient induction of mitotic catastrophe. Nevertheless, there is no evidence directly demonstrating the association of complex DSBs with efficient induction of mitotic catastrophe and further research is required.

There is some controversy as to whether mitotic catastrophe is a distinct mode of cell death. In the field of radiation biology, mitotic catastrophe is considered a major mode of cell death induced by ionizing irradiation that is distinct from other mechanisms of clonogenic cell death, including apoptosis and necrosis^39^. Here, we describe mitotic catastrophe as cell death distinct from apoptosis. Meanwhile, others argue that mitotic catastrophe may not be a distinct mode of cell death, but rather a process that precedes cell death (including apoptosis and necrosis)^43^. Further studies should examine the mechanism underlying induction of mitotic catastrophe by radiation and chemotherapy.

In clinical carbon ion radiotherapy, high single-dose irradiation is often used with a hypo-fractionated treatment schedule^1^. Recent studies suggest that stereotactic ablative X-ray radiotherapy using high single-dose irradiation contributes to the efficient induction of antitumor immunity^44^. From this point of view, it is possible that carbon ion radiotherapy induces antitumor immunity more efficiently than conventional X-ray radiotherapy using 2-Gy-per-day fractionation. Therefore, the effect on induction of antitumor immunity should be taken into account when exploring the cell killing mechanism of carbon ions. There is little knowledge regarding this issue and further studies are needed.

Morphological observation of DAPI-stained nuclei has been used to assess mitotic catastrophe, apoptosis, and senescence by both us and others^28^,^29^,^30^,^31^. Nevertheless, additional assessment of the type of clonogenic cell death using other methods, such as flow cytometry, would further improve the robustness of the results. We were unable to conduct such experiments in this study because carbon ions are a limited experimental resource.

The present study has one limitation. We used a physical dose of 4 Gy for carbon ions and X-rays to assess the mode of cell death. In general, 4 Gy carbon ions have a stronger cell killing effect than 4 Gy X-rays. Therefore, the comparison of cell death after treatment with carbon ions and that after treatment with X-rays using the same physical dose of 4 Gy might have confounded the results. Usage of the isoeffective dose can provide more solid results. The isoeffective dose varies according to the survival endpoint. Therefore, isoeffective doses for multiple survival endpoints should be used for assessment. In addition, the isoeffective dose for a given survival endpoint varies among cell lines because the relative biological effectiveness of carbon ions over X-rays varies among cell lines. In this study, it was not possible to test various isoeffective doses due to the limited experimental resources for carbon ion irradiation. Therefore, we employed the same physical dose of 4 Gy. Future experiments using isoeffective doses for multiple survival endpoints will further clarify this issue.

In summary, the data presented herein indicate that the correlation of sensitivity to carbon ions and cisplatin is weaker than that of sensitivity to X-rays and cisplatin, and that mitotic catastrophe is a putative driver for the weak correlation between carbon ion and cisplatin sensitivities. The results are helpful as biological basis to understand the potentially shared mechanism among these treatments, although further investigation is mandatory to elucidate the clinical efficacy of carbon ions and cisplatin combination.

## Methods

### Materials

Twenty human cancer cell lines of various origin (Supplementary Table 5) were cultured in RPMI-1640 (Sigma-Aldrich, St. Louis, MO, USA) supplemented with 10% fetal bovine serum (Life Technologies, Carlsbad, CA, USA). Cisplatin and staurosporine were obtained from Sigma-Aldrich.

### Irradiation

X-ray irradiation was performed using a Faxitron RX-650 (100 kVp, 1.14 Gy/min; Faxitron Bioptics, Tucson, AZ, USA). Carbon ion irradiation was performed at the Gunma University Heavy Ion Medical Center using the beam specifications used in a clinical setting (290 MeV/nucleon and an average linear energy transfer at the center of a 6 cm spread-out Bragg peak of approximately 50 keV/μm). Carbon ion beams were delivered in a vertical direction so that cells on culture plates received an even dose.

### Clonogenic survival assay

Cells were seeded into 6 well plates and irradiated with X-rays or carbon ions, or exposed to cisplatin for 24 h. After incubation for 10 days, cells were fixed with methanol and stained with crystal violet. Colonies comprising at least 50 cells were counted. The surviving fraction was normalized to that of the corresponding controls. Survival curves were generated by fitting the surviving fraction to a linear-quadratic model: SF = exp(−αD −βD^2^), where SF is the surviving fraction and D is the dose. The D_10_ values were calculated by solving the resulting equations for a survival of 10%^45^.

### Evaluation of clonogenic cell death

Cells were grown on glass coverslips and received the treatment of interest. Seventy-two hours later, cells were stained with DAPI. Cells were examined under a fluorescence microscope (Eclipse Ni, Nikon, Tokyo, Japan) at ×100 magnification, and the mode of clonogenic cell death was determined according to characteristic nuclear morphologies. Apoptosis was determined according to characteristic nuclear morphology: the presence of apoptotic bodies, nuclear condensation, and fragmentation^27^. Cells containing nuclei with two or more distinct lobes were scored as positive for mitotic catastrophe^28^,^29^,^30^. Cells containing nuclei with senescence-associated heterochromatic foci were scored as positive for senescence^31^. At least 300 cells were evaluated for each experimental condition.

### Annexin-V staining assay

Cells were treated with X-rays, carbon ions, or staurosporine and then stained with AV-FLUOS and PI using an Annexin-V-FLUOS Staining Kit (Roche Diagnostics, Indianapolis, IN, USA), according to the manufacturer′s protocol. Stained cells were analyzed on a Guava easyCyte HT (Millipore, Billerica, MA, USA).

### Statistical analysis

Statistical analysis was performed using SigmaPlot 12.0 (Hulinks, Tokyo, Japan). Correlations were evaluated using Spearman’s Rank Order test. Statistical significance was evaluated using an unpaired two-tailed *t*-test. *P *< 0.05 was considered statistically significant.

## Additional Information

**How to cite this article**: Kobayashi, D. *et al*. Mitotic catastrophe is a putative mechanism underlying the weak correlation between sensitivity to carbon ions and cisplatin. *Sci. Rep.* **7**, 40588; doi: 10.1038/srep40588 (2017).

**Publisher's note:** Springer Nature remains neutral with regard to jurisdictional claims in published maps and institutional affiliations.

## Supplementary Material

Supplementary Information
