# Supplementary material for: Mitotic catastrophe is a putative mechanism underlying the weak correlation between sensitivity to carbon ions and cisplatin
Source: Sci Rep. 2017 Jan 16;7:40588. doi: 10.1038/srep40588 (PMC5238371; doi:10.1038/srep40588)
Supplement: Supplementary Information [file srep40588-s1.pdf]

Supplementary information

Title: Mitotic catastrophe is a putative mechanism underlying the weak correlation between sensitivity to carbon ions and cisplatin

Authors: Daijiro Kobayashi[1], Takahiro Oike[1,2]*, Atsushi Shibata[3], Atsuko Niimi[4], Yoshiki Kubota[5], Makoto Sakai[5], Napapat Amornwhichet[1,6], Yuya Yoshimoto[1], Yoshihiko Hagiwara[3], Yuka Kimura[1], Yuka Hirota[1], Hiro Sato[1], Mayu Isono[5], Yukari Yoshida[5], Takashi Kohno[2], Tatsuya Ohno[5], and Takashi Nakano[1,5]

Affiliations: [1]Department of Radiation Oncology, Gunma University Graduate School of Medicine, Maebashi, Gunma, Japan; [2]Division of Genome Biology, National Cancer Center Research Institute, Chuo-ku, Tokyo, Japan; [3]Advanced Scientific Research Leaders Development Unit, Gunma University, Maebashi, Gunma, Japan; [4]Research Program for Heavy Ion Therapy, Division of Integrated Oncology Research, Gunma University Initiative for Advanced Research, Maebashi, Gunma, Japan; [5]Gunma University Heavy Ion Medical Center, Maebashi, Gunma, Japan; [6]Department of Radiology, Chulalongkorn University, Pathumwan, Bangkok, Thailand.

***Corresponding author:**
Takahiro Oike, MD, PhD
Assistant Professor
Department of Radiation Oncology, Gunma University Graduate School of Medicine
3-39-22, Showa-machi, Maebashi, Gunma 371-8511, Japan.
Tel: +81-027-220-8383
Fax: +81-027-220-8397
E-mail: oiketakahiro@gmail.com

Supplementary Table 1. Summary of the studies on synergistic cell killing effect between X-rays or carbon ions and cisplatin in human cancer cells.

Radiation	Cell line	Synergistic?	References
X-rays	AE7	yes	11
	AN3	yes	
	ECC1	yes	
	HEC1A	yes	
	HEC1B	yes	
	SKUT1B	yes	
	HeLa	yes	12
	C33A	yes	
	A549	yes	13
	CaSki	yes	
	SW-1573	yes	14
	WiDr	yes	8
	A2780cp	yes	15
	A2780	no	
	DU-145	controversial	16
	19 primary tumors	no	17
	HeLa	no	18
	CaSki	no	
	MRI-186	no	
	LX-I	no	
	A549	no	19
	HepG2	no	20
	OV-1063	no	21
	TE-2	no	9
	U87	no	10
Carbon ion	MDA-MB-231	yes	7
	WiDr	no	8
	TE-2	no	9
	U87	no	10

See "Supplementary Methods" for the detailed methods for litereature search.

Supplementary Table 2. Parameters for X-ray and carbon ion sensitivity.

Cell line	X-rays						Carbon ion						RBE	
	D10		α		β		D10		α		β			
	Mean	SD	Mean	SD	Mean	SD	Mean	SD	Mean	SD	Mean	SD	Mean	SD
H1299	10.9	0.76	0.0024	0.026	0.019	0.0019	3.9	0.12	0.079	0.034	0.13	0.018	2.8	0.075
H157	6.7	0.27	0.051	0.079	0.043	0.010	3.3	0.14	0.63	0.060	0.020	0.022	2.0	0.13
LK2	6.7	0.28	0.066	0.031	0.041	0.0049	3.3	0.075	0.40	0.095	0.094	0.029	2.1	0.042
H1703	9.2	0.66	0.16	0.022	0.010	0.0033	4.3	0.37	0.31	0.12	0.055	0.033	2.1	0.21
HCT15	6.3	0.11	0.13	0.017	0.039	0.0029	2.3	0.061	0.75	0.15	0.10	0.028	2.7	0.17
II-18	7.4	0.78	0.048	0.26	0.036	0.035	4.1	0.56	0.42	0.062	0.035	0.0069	1.8	0.21
A549	6.6	0.67	0.30	0.11	0.0076	0.016	4.0	0.21	0.25	0.079	0.084	0.020	1.7	0.036
WiDr	6.9	0.11	0.0047	0.031	0.048	0.0051	3.3	0.10	0.33	0.25	0.12	0.084	2.1	0.12
HCT116	4.0	0.13	0.39	0.15	0.044	0.035	1.6	0.19	1.3	0.18	0.088	0.048	2.6	0.26
A427	5.3	0.43	0.29	0.049	0.028	0.010	2.1	0.14	1.0	0.21	0.033	0.067	2.6	0.23
KYSE70	6.8	0.26	-0.018	0.085	0.053	0.019	2.5	0.10	-0.33	0.11	0.51	0.055	2.8	0.19
H1975	4.7	0.26	0.44	0.070	0.010	0.0088	2.4	0.26	0.20	0.21	0.32	0.079	2.0	0.15
HCC827	5.5	0.22	0.29	0.071	0.024	0.012	2.6	0.064	0.60	0.10	0.11	0.029	2.1	0.124
H522	6.0	0.29	0.11	0.063	0.046	0.012	2.1	0.08	0.97	0.036	0.076	0.0049	2.9	0.11
TE2	2.1	0.10	0.88	0.13	0.093	0.047	0.94	0.13	1.5	0.56	1.1	0.85	2.3	0.25
H460	4.8	0.20	0.23	0.017	0.052	0.0038	1.8	0.061	0.82	0.11	0.25	0.078	2.6	0.079
HeLa	6.7	0.19	0.19	0.080	0.023	0.014	3.1	0.079	0.26	0.19	0.15	0.065	2.1	0.10
H1650	4.8	1.00	-0.10	0.20	0.12	0.037	2.7	0.16	0.56	0.10	0.10	0.024	1.8	0.17
Ma-24	3.9	0.35	0.22	0.27	0.11	0.089	2.8	0.14	0.34	0.15	0.17	0.056	1.4	0.29
PC9	3.4	0.13	0.64	0.14	0.011	0.026	2.5	0.19	0.32	0.28	0.25	0.12	1.4	0.18

RBE, relative biological effectiveness; SD, standard deviation.

Supplementary Table 3. Correlation between X-ray- and cisplatin-sensitivity.

Index	X-rays	Cisplatin		
		0.2 μM	1 μM	IC50
R value	SF2Gy	0.59	0.63	0.68
	SF4Gy	0.70	0.76	0.76
	SF6Gy	0.62	0.65	0.61
	SF8Gy	0.41	0.68	0.60
	D10	0.70	0.76	0.74
P value	SF2Gy	6.0E-3	2.7E-3	9.6E-4
	SF4Gy	4.1E-4	2.0E-7	2.0E-7
	SF6Gy	4.5E-3	2.8E-3	5.5E-3
	SF8Gy	0.035	1.4E-3	6.2E-3
	D10	4.6E-4	2.0E-7	8.1E-5

SFXGy indicates the surviving fraction at X Gy.

Supplementary Table 4. Correlation between carbon ion- and cisplatin-sensitivity.

Index	Carbon ion	Cisplatin		
		0.2 µM	1 µM	IC50
R value	SF1Gy	0.44	0.47	0.46
	SF2Gy	0.35	0.43	0.39
	SF3Gy	0.30	0.55	0.53
	SF4Gy	0.51	0.53	0.44
	D10	0.45	0.57	0.49
P value	SF1Gy	0.049	0.037	0.042
	SF2Gy	0.13	0.056	0.089
	SF3Gy	0.23	0.018	0.024
	SF4Gy	0.031	0.024	0.068
	D10	0.044	9.2E-3	0.027

SFXGy indicates the surviving fraction at X Gy.

Supplementary Table 5. Cell lines used in the present study.

Cell line	Cancer type	Histology	Obtained from
KYSE70	Esophageal cancer	Squamous cell carcinoma	Health Science Research Resources Bank (Osaka, Japan)
TE2	Esophageal cancer	Squamous cell carcinoma	University of Tohoku Cell Bank (Sendai, Japan)
A427	Non-small cell lung carcinoma	Adenocarcinoma	ATCC (Manassas, VA, USA)
A549	Non-small cell lung carcinoma	Adenocarcinoma	ATCC (Manassas, VA, USA)
H522	Non-small cell lung carcinoma	Adenocarcinoma	ATCC (Manassas, VA, USA)
H1650	Non-small cell lung carcinoma	Adenocarcinoma	ATCC (Manassas, VA, USA)
H1703	Non-small cell lung carcinoma	Adenocarcinoma	ATCC (Manassas, VA, USA)
H1975	Non-small cell lung carcinoma	Adenocarcinoma	ATCC (Manassas, VA, USA)
HCC827	Non-small cell lung carcinoma	Adenocarcinoma	ATCC (Manassas, VA, USA)
II-18	Non-small cell lung carcinoma	Adenocarcinoma	JCRB Cell Bank (Osaka, Japan)
Ma-24	Non-small cell lung carcinoma	Adenocarcinoma	Dr. Shimizu (Tokushima University, Tokushima, Japan)
PC9	Non-small cell lung carcinoma	Adenocarcinoma	Dr. Kato (Tokyo Medical Collage, Tokyo, Japan)
H157	Non-small cell lung carcinoma	Squamous cell carcinoma	Dr. Harris (National Institute of Health, MD, USA)
LK2	Non-small cell lung carcinoma	Squamous cell carcinoma	JCRB Cell Bank (Osaka, Japan)
H460	Non-small cell lung carcinoma	Large cell carcinoma	ATCC (Manassas, VA, USA)
H1299	Non-small cell lung carcinoma	Large cell carcinoma	ATCC (Manassas, VA, USA)
HCT15	Colorectal cancer	Adenocarcinoma	ATCC (Manassas, VA, USA)
HCT116	Colorectal cancer	Adenocarcinoma	Dr. Vogelstein (Johns Hopkins University, MD, USA)
WiDr	Colorectal cancer	Adenocarcinoma	ATCC (Manassas, VA, USA)
HeLa	Uterine cervical cancer	Adenocarcinoma	ATCC (Manassas, VA, USA)

Supplementary Figure 1

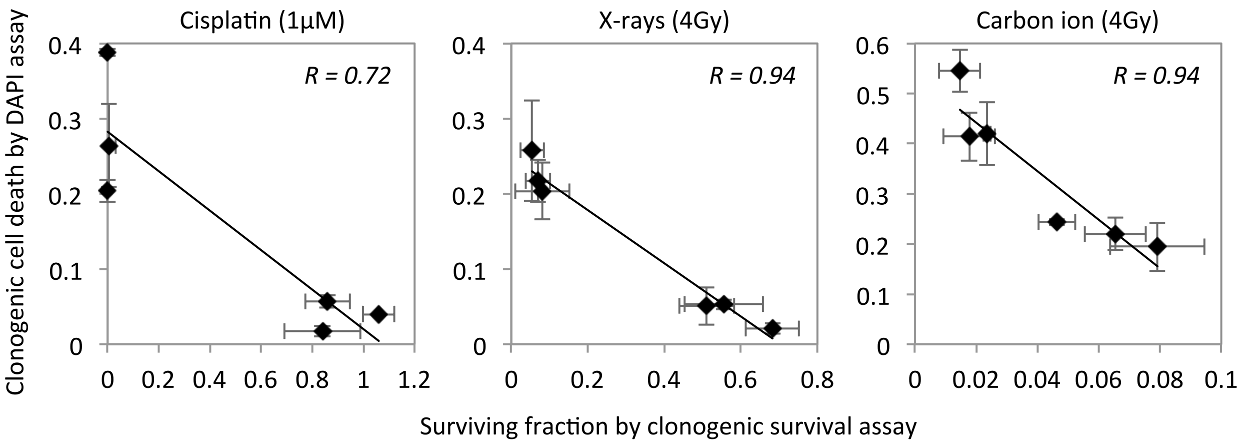

Supplementary Figure 1. Correlation between the total amount clonogenic cell death (apoptosis, mitotic catastrophe and senescence) as assessed by the DAPI staining assay and the surviving fraction as assessed by the clonogenic survival assays in PC9, Ma-24, H1650, H1299, H157, and LK2 cells, as assessed by Spearman Rank Order test. Note that the data for the clonogenic survival assay and those for the DAPI staining assay are the same as in Figure 1 and 2, and Figure 4b, respectively, but now in the different context.

Supplementary Figure 2

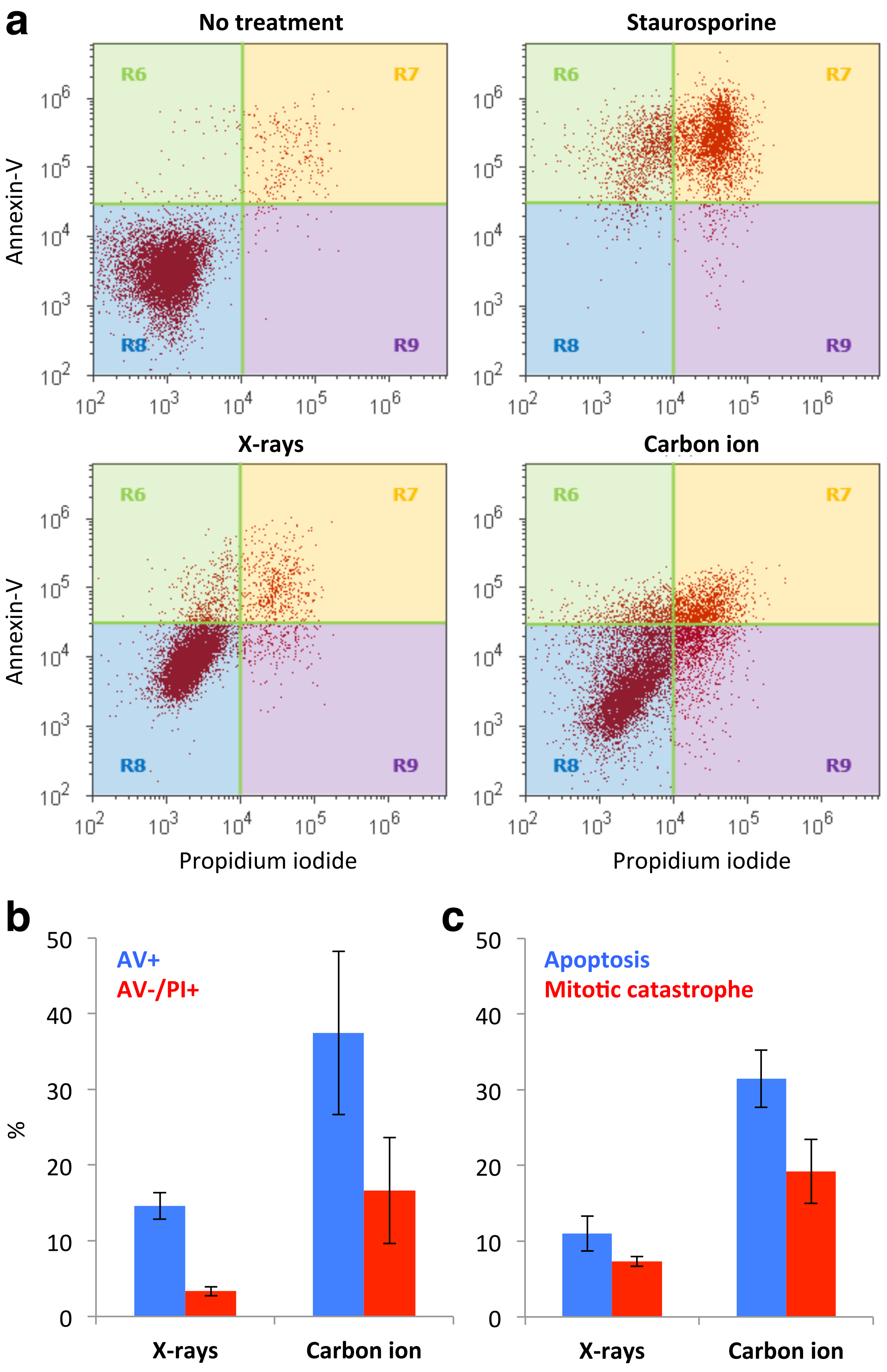

Supplementary Figure 2. Cell death induced by X-ray or carbon ion irradiation as assessed by flow cytometry or the DAPI staining assay. PC9 cells were treated with X-rays (4 Gy for 72 h), carbon ions (4 Gy for 72 h), or staurosporine (1 μM for 24 h), followed by the staining with AV and PI or DAPI. The AV/PI-stained cells were analyzed by flow cytometry, while the DAPI stained cells were analyzed for nuclear morphologies representing apoptosis and mitotic catastrophe (see "Materials and Methods" for definitions). **(a)** Representative flow cytometry plots. High AV-positivity in staurosporine-treated cells indicates the robustness of the assay (Supplementary references 1, 2). **(b)** Percentages of AV-positive and AV-negative/PI-positive cells determined by flow cytometry. **(c)** Percentages of apoptosis and mitotic catastrophe determined by the DAPI staining assay. Note that the data for the DAPI staining assay are the same as in Figure 4b but now in the different context.

Supplementary Methods

Literature search: synergistic cell killing by X-rays or carbon ions plus cisplatin.

On January 15, 2016, two authors (D. K. and T. Oike) performed independent literature searches in PubMed using the search term "cisplatin AND radiosensitization" and obtained 147 hits. A full text screen for all available papers identified 15 that used clonogenic survival assays to examine the cell killing effects of X-rays and/or carbon ions combined with cisplatin in human cancer cells. The presence or absence of synergistic cell killing effect was analyzed: cell killing effects classified as "supra-additive", "independently toxic", and "radiosensitizing" were considered synergistic, whereas effects described as "additive" were considered non-synergistic. The results are summarized in Supplementary Table 1.

Supplementary References

1. Nakagawa, T. *et al*. Caspase-12 mediates endoplasmic-reticulum-specific apoptosis and cytotoxicity by amyloid-beta. *Nature* **403**, 98–103 (2000)
2. Takayama, S. *et al*. Cloning and functional analysis of BAG-1: a novel Bcl-2-binding protein with anti-cell death activity. *Cell* **80**, 279–284 (1995)
